# Osteoprotegerin and coronary artery disease in type 2 diabetic patients with microalbuminuria

**DOI:** 10.1186/1475-2840-10-70

**Published:** 2011-07-29

**Authors:** Henrik Reinhard, Mads Nybo, Peter R Hansen, Niels Wiinberg, Andreas Kjær, Claus L Petersen, Kaj Winther, Hans-Henrik Parving, Lars M Rasmussen, Peter Rossing, Peter K Jacobsen

**Affiliations:** 1 Steno Diabetes Center, Gentofte, Denmark; 2 Department of Clinical Biochemistry and Pharmacology, Odense University Hospital, Odense, Denmark; 3 Department of Cardiology, Gentofte University Hospital, Gentofte, Denmark; 4 Department of Clinical Physiology and Nuclear Medicine, Frederiksberg University Hospital Frederiksberg, Denmark; 5 Department of Clinical Physiology, Nuclear Medicine & PET and Cluster for Molecular Imaging, University Hospital of Copenhagen, Copenhagen, Denmark; 6 Department of Clinical Biochemistry, Frederiksberg University Hospital, Frederiksberg, Denmark; 7 Department of Medical Endocrinology, University Hospital of Copenhagen, Copenhagen, Denmark; 8 Faculty of Health Science, Aarhus University, Aarhus, Denmark; 9 The Heart Centre, University Hospital of Copenhagen, Copenhagen, Denmark

## Abstract

**Objective:**

Plasma osteoprotegerin (P-OPG) is an independent predictor of cardiovascular disease in diabetic and other populations. OPG is a bone-related glycopeptide produced by vascular smooth muscle cells and increased P-OPG may reflect arterial damage. We investigated the correlation between P-OPG and coronary artery disease (CAD) in asymptomatic type 2 diabetic patients with microalbuminuria.

**Methods:**

P-OPG was measured in 200 asymptomatic diabetic patients without known cardiac disease. Patients with P-NT-proBNP >45.2 ng/l and/or coronary calcium score (CCS) ≥400 were stratified as high risk of CAD (n = 133), and all other patients as low risk patients (n = 67). High risk patients were examined by myocardial perfusion imaging (MPI; n = 109), and/or CT-angiography (n = 20), and/or coronary angiography (CAG; n = 86). Significant CAD was defined by presence of significant myocardial perfusion defects at MPI and/or >70% coronary artery stenosis at CAG.

**Results:**

Significant CAD was demonstrated in 70 of the high risk patients and of these 23 patients had >70% coronary artery stenosis at CAG. Among high risk patients, increased P-OPG was an independent predictor of significant CAD (adjusted odds ratio [CI] 3.11 [1.01-19.54] and 3.03 [1.00-9.18] for second and third tertile vs.first tertile P-OPG, respectively) and remained so after adjustments for NT-proBNP and CCS. High P-OPG was also associated with presence of >70% coronary artery stenosis(adjusted odds ratio 14.20 [1.35-148.92] for third vs. first tertile P-OPG), and 91% of patients with low (first tertile) P-OPG did not have >70% coronary artery stenosis.

**Conclusions:**

Elevated P-OPG is an independent predictor of the presence of CAD in asymptomatic type 2 diabetic patients with microalbuminuria.

## Introduction

Coronary artery disease (CAD) is the most important determinant of the excessive morbidity and mortality in type 2 diabetic patients, especially in patients with albuminuria[1]. Plasma osteoprotegerin (P-OPG) is a promising predictor of cardiovascular disease (CVD) in high risk diabetic populations, as well as in other populations [2-5]. Osteoprotegerin is a member of the tumor necrosis factor (TNF) receptor superfamily acting as a soluble decoy receptor for the receptor activator of nuclear factor-κβ ligand (RANKL) and TNF-related apoptosis-inducing ligand (TRAIL) preventing osteoclast activation and bone resorption, and participating in immune regulation and cell survival [6,7]. OPG mRNA has been detected in a variety of human tissues, including the lung, heart, and kidney[6]. Indeed, OPG is present in the arterial wall and increased P-OPG has been suggested to reflect the increased OPG content in atherosclerotic arterial tissues[8]. OPG is up-regulated in calcified coronary plaques and is associated with coronary angiographic disease severity and cardiovascular events independent of conventional risk factors [9,10]. Patients with diabetes have elevated P-OPG [11] and in uncomplicated type 2 diabetic patients that were followed for 18 months, P-OPG predicted cardiovascular events but was also associated with subclinical coronary calcification burden, as measured by non-invasive CT-determined coronary calcium score (CCS)[4]. We have recently shown that OPG is a strong and independent predictor of cardiovascular (CVD) morbidity and mortality in type 2 diabetic patients in a study with 17 years of follow-up[3]. The underlying causes of CVD in patients with elevated P-OPG levels are not known and the putative role of asymptomatic CAD in this regard is not clear. Consequently, we evaluated if P-OPG could serve as a biomarker for subclinical CAD in asymptomatic type 2 diabetic patients with microalbuminuria.

## Methods

### Patient cohort and investigations

In a cross-sectional study at Steno Diabetes Center, we identified from January 2007 to February 2008 a consecutive cohort of 200 type 2 diabetic patients with microalbuminuria but without prior heart disease and with normal P-creatinine. All patients received intensive multifactorial intervention aimed at improved glucose, lipid, and blood pressure control, as well as antithrombotic therapy and lifestyle modification according to international treatment guidelines[12]. The design and a selection of clinical measurements of the study have previously been described[13]. In brief, P-NT-proBNP was analysed by an established immunoassay[14]. Tests for autonomic neuropathy, heart rate variability assessed by the expiration-inspiration variation of the heart rate and somatic nerve function (vibratory perception threshold) evaluated by biothesiometry, were performed as reported previously[13]. Carotid artery intima media thickness (CIMT) was measured at the posterior wall of the common carotid artery 20 mm proximal to the bifurcation bilaterally (Siemens Acuson Cypress ultrasound scanner) and calculated as the mean of CIMT on both sides. Furthermore, systolic blood pressures in the ankle and big toe were measured on both legs by strain gauge technique and the lowest ankle and big toe pressures were recorded and calculated as ankle-brachial (ABI) and toe-brachial (TBI) blood pressure index[15]. Agaston CCS was measured during a single breath hold using a 16 multidetector-row CT scanner with 3 mm slice thickness (Philips Precedence MX 8000 IDT 16 slice, Philips Medical Systems, Best, The Netherlands)[16].

### Risk stratification scheme and assessment of CAD

CCS ≥400 was used to designate high risk diabetic patients as recently reported [17]. Patients were stratified into high risk and low risk groups based on elevated P-NT-proBNP and elevated CCS as follows: 1) P-NT-proBNP >45.2 ng/l or CCS ≥400 = high risk patients (n = 133); the P-NT-proBNP cut-off was the median value among the first 50 patients examined in the study and this cut-off was in agreement with our earlier findings[14], 2) P-NT-proBNP ≤45.2 ng/l and CCS <400 = low risk patients (n = 67). High risk patients were examined according to the following algorithm: 1) Patients with P-NT-proBNP >45.2 ng/l underwent myocardial perfusion imaging (MPI). Patients with abnormal MPI (n = 55) or CCS >100 (n = 29) were referred for coronary angiography (CAG); 2) Patients with P-NT-proBNP ≤45.2 ng/l and CCS 400-1000 underwent CT angiography (CTA) (n = 20; CTA was only used in patients with CCS 400-1000 since severe coronary artery calcifications (CCS > 1000) compromise the validity of CTA)[18]. Patients with abnormal CTA were referred for CAG (n = 15); and 3) Patients with P-NT-proBNP ≤45.2 ng/l and CCS >1000 underwent MPI (n = 9). Patients with abnormal MPI (n = 6) were referred for CAG.

Significant CAD was defined as the presence of significant myocardial perfusion defects on MPI, and/or >70% stenosis of one or more major epicardial coronary arteries at CAG as determined by quantitative analysis (Phillips Medical Systems). The correlations between P-NT-proBNP, CTA, MPI and CAG have been reported previously[13].

### Measurement of P-OPG

Blood samples for determination of OPG were collected in EDTA-citrated tubes, centrifuged and the isolated plasma was stored at -80°C until analysis within two years from sampling. P-OPG was measured in random order by a sandwich ELISA assay using commercially available antibodies (R&D Systems, Minneapolis, MN, USA), as previously described[19]. Briefly, mouse antihuman OPG was used as capture antibody and a biotinylated goat anti-human OPG for detection. Recombinant human OPG was used for calibration and the analytical range of the assay was 62.5-4.000 pg/ml. The intra-assay coefficient of variation was 3% and the inter-assay variation less than 8% in duplicate measurements as previously reported[2].

The study was approved by the local ethics committee and all patients gave written informed consent.

### Statistical analyses

We investigated the associations of P-OPG with age, sex and risk factors in both univariate and multivariate linear regression analyses. Risk factors included in the analyses were diabetes duration, HbA_1c _levels, systolic blood pressure, P-total cholesterol, P-creatinine, heart rate variation, vibration threshold, presence of retinopathy, level of microalbuminuria, P-NT-proBNP, peripheral systolic blood pressure in the toe, CIMT and CCS. Furthermore, all patients were divided into groups of patients with or without significant CAD as determined by MPI and/or CAG. In most of our analyses, we included all patients and thereby assumed that low risk patients (P-NT-proBNP ≤45.2 ng/l and CCS <400, see above) were without significant CAD. We did, however, also examine if OPG was predictive of CAD within the high risk group alone, where all patients were investigated for CAD by CTA, MPI and/or CAG. Comparisons between groups were performed by an unpaired Student's t-test or the Pearson Chi-square test as appropriate. Data were expressed as means and standard deviation (SD), except for non-normally distributed variables, which were log10-transformed before analysis and are reported as medians (interquartile [IQ] range). As CCS was highly skewed with values of zero, log10 (CCS + 1) was used for analysis. Finally, the associations between significant CAD and high, intermediate and low P-OPG divided in tertiles or natural log-transformed P-OPG, respectively, were assessed by multivariate logistic regression models and expressed as odds ratios (ORs). Covariate adjustments were made for age, sex and variables associated with significant CAD in the univariate analysis (p < 0.05), including P-total cholesterol, P-creatinine, peripheral systolic blood pressure, vibratory perception threshold and heart rate variability unless otherwise stated. In addition, the predictive accuracy of the covariate-adjusted models with and without inclusion of P-OPG were compared by generating receiver operating characteristic (ROC) curves for endpoints and the areas under the ROC curves (AUCs) were calculated. All data were analyzed by using statistical package for social sciences (SPSS) version 14 for windows, and a p-value less than 0.05 was considered as statistically significant.

## Results

### Clinical characteristics

The clinical characteristics of all patients, low vs. high risk patients, and patients with or without significant CAD, respectively, are summarized in Table 1.

**Table 1:** The clinical characteristics of all patients, low vs. high risk patients and patients with significant coronary artery disease (CAD; abnormal MPI and/or stenosis on coronary angiography).

	All patients (n = 200)	Low-risk patients (n = 67)	High-risk patients (n = 133)	p-values^1^	Patients without sign. CAD (n = 63)	Patients with sign. CAD (n = 70)	p-values^2^
Sex no. (male%)	152 (76)	50(75)	102 (77)	0.747	14 (20)	56 (80)	0.33
Age (years)	59 (9)	53 (10)	61 (6)	0.001	57 (9)	62 (6)	0.001
Duration of diabetes (years)	13 (7)	10 (7)	14 (7)	0.001	12 (7)	15 (7)	0.006
BMI (kg/m^2^)	32.6 (5.8)	32.9 (6.0)	32.4 (5.7)	0.592	32.3 (5.8)	33.1 (5.7)	0.32
HbA_1c _(%)	7.9 (1.3)	8.0 (1.3)	7.8 (1.4)	0.194	7.8 (1.3)	7.8 (1.5)	0.80
Urinary albumin excretion rate (mg/24h)*	103 (39 - 230)	105 (44 - 194)	97 (38 - 97)	0.814	97 (43 - 194)	133 (38 - 491)	0.22
P-creatinine (μmol/l)	76 (18)	72 (17)	79 (19)	0.007	73 (17)	82 (20)	0.001
Systolic blood pressure (mmHg)	130 (17)	130 (16)	130 (18)	0.953	129 (17)	132 (18)	0.38
Total cholesterol (mmol/l)	3.9 (0.9)	4.1 (1.0)	3.8 (0.9)	0.041	4.0 (1.0)	3.8 (0.9)	0.08
Vibratory perception threshold mV - mean of both sides	33 (15)	28 (14)	36 (14)	0.001	30 (14)	39 (13)	0.001
Heart rate variation during deep breathing (bpm)*	7 (4.5-11.5)	9 (7-14)	6 (4-10)	0.001	8 (5-12)	6 (4-10)	0.013
Retinopathy no. (%)	120 (60)	28 (42)	92 (69)	0.001	71(55)	49 (70)	0.034
Oral antidiabetic medication no. (%)	170 (85)	57 (85)	113 (85)	0.98	111(85)	59 (84)	0.83
Insulin treatment no. (%)	124 (62)	38 (57)	86 (65)	0.28	77(59)	47 (67)	0.27
RAAS blockade no. (%)	196 (98)	65 (97)	131 (98)	0.48	127 (98)	69 (99)	0.67
Statin therapy no. (%)	189 (95)	62 (93)	127 (95)	0.39	122 (94)	67 (96)	0.58
Aspirin therapy no. (%)	183 (92)	58 (87)	125 (94)	0.076	119 (92)	69 (99)	0.98
Beta-blocker therapy no. (%)	27 (14)	2 (3)	25 (19)	0.002	12 (9)	15 (21)	0.016
Calcium channel blockers no. (%)	80 (40)	21 (31)	59 (44)	0.076	47 (36)	33 (47)	0.13
Use of diuretics no. (%)	128 (64)	35 (52)	93 (70)	0.014	82(63)	46(66)	0.71
Current smoker no. (%)	59 (30)	18 (27)	41 (31)	0.56	36(28)	23 (33)	0.45
NT-proBNP (ng/l) *	48.7 (18.6-95.0)	15.3 (9.3-26.3)	77.1 (48.7-141.7)	nr	30.1 (13.4-77.1)	76.7 (52.8-139.5)	0.001
Coronary Calcium Score*	183 (6-604)	7 (0-104)	417 (80-963)	nr	71 (1-272)	748 (177-1518)	0.001

Data are expressed as means (SD) or * medians (interquartile range).

high-risk patients = patients with plasma NT-proBNP levels >45.2 ng/l or plasma NT-proBNP levels ≤45.2 ng/l and CCS ≥400, all other low-risk patients.

nr = not relevant.

p-values reflect comparison 1) between high- and low risk patients and 2) patients with or without significant CAD.

### P-OPG and established cardiovascular risk factors

Among the 200 type 2 diabetic patients, median (IQ range) P-OPG was 1962 (1589-2409) pg/ml. P-OPG was positively associated with age (r = 0.26, p < 0.001), diabetes duration (r = 0.18, p = 0.010), vibration threshold (r = 0.24, p < 0.001), and P-NT-proBNP (r = 0.27, p < 0.001), while inversely related to increased heart rate variation (r = -0.20, p = 0.007). P-OPG differed in patients with or without retinopathy (2248 pg/ml vs. 1886, p = 0.002), but not between men and women (p = 0.12). P-OPG was not associated with HbA1c levels (p = 0.17), systolic blood pressure (p = 0.52), P-total cholesterol (p = 0.21), P-creatinine (p = 0.43), microalbuminuria (p = 0.11), peripheral systolic blood pressure in the toe (p = 0.11), or CIMT (p = 0.36), respectively. Interestingly, however, P-OPG was associated with CCS (r = 0.24, p = 0.001). Adjusted OR of CCS ≥400 in patients with P-OPG in the upper vs. lower tertile was 2.38 (0.996-5.69), p = 0.051 (Table 2). In addition, when adding other CCS-associated variables (diabetes duration, vibration threshold, retinopathy and systolic blood pressure in the big toe), high (third tertile) P-OPG levels remained an independent predictor of CCS ≥400 with OR 2.54 (1.01-6.37), p = 0.048.

**Table 2:** The relations between plasma OPG and the prevalence of coronary atherosclerosis as defined by CCS ≥400.

Parameter	OR	95% CI	p-value
Study population as a whole (n = 200)	Coronary atherosclerosis		
Unadjusted OPG (2^rd ^tertile vs. 1^st^)	2.39	1.09-5.25	0.03
Unadjusted OPG (3^rd ^tertile vs. 1^st^)	3.36	1.54-7.34	0.002
Adjusted OPG (2^rd ^tertile vs. 1^st^)	2.50	1.08-5.78	0.032
Adjusted OPG (3^rd ^tertile vs. 1^st^)	2.62	1.12-6.11	0.0026

Odds ratios (ORs) were assessed by logistic regression in univariate and multivariable analyses (adjusted for sex and age).

Patients were also divided according to P-OPG tertiles (<1679, 1679-2199, and >2199 pg/ml, respectively). The patients with high (third tertile) P-OPG levels were older (p < 0.001), had a longer diabetes duration (p = 0.006), more retinopathy (p = 0.003) and higher P-NT-proBNP (p = 0.01). Furthermore, these patients had higher vibration threshold (p < 0.001) and lower heart rate variation (p = 0.024).

### P-OPG and subclinical coronary artery disease

In 70 of 133 (53%) high risk patients, significant CAD was demonstrated by MPI and/or CAG, corresponding to 35% (70/200) of the total cohort. In addition, among patients with significant CAD, 23 patients had significant (>70%) coronary artery stenosis as defined by CAG.

**Figure 1:**
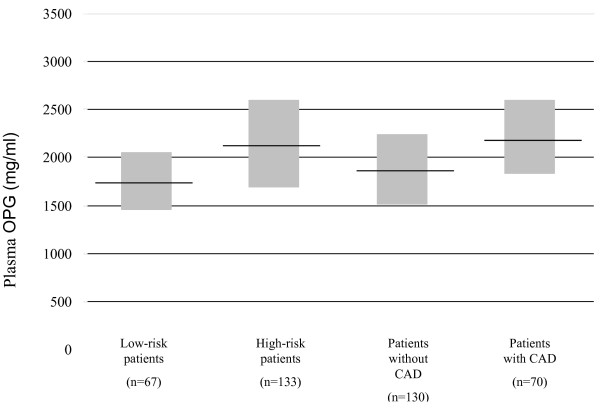
**Diagram showing medians (interquartile range) of plasma osteoprogeterin in low vs. high-risk patients (p < 0.001) and patients with or with significant coronary artery disease (CAD; abnormal MPI and/or stenosis on coronary angiography, (p < 0.001)**. High risk patients = patients with plasma NT-proBNP levels >45.2 ng/l and/or CCS ≥400, all other low risk patients.

In the looking of the whole cohort, P-OPG was higher in patients with significant CAD (Figure 1). Furthermore, in a logistic regression model, intermediate and high (second and third tertile values) P-OPG compared to low (first tertile values) were strongly associated with significant CAD (Table 3). In addition, when adding P-NT-proBNP and CCS to the adjusted model, second and third tertile P-OPG concentrations remained independently predictive of significant CAD with OR of 3.63 (1.24-10.60) and 3.95 (1.36-11.52), respectively. This pattern was also found after exclusion of the low risk patients that did not undergo cardiac examination by CTA, MPI and/or CAG, i.e., OR 3.11 (1.01-19.54) and 3.03 (1.00-9.18), respectively. In addition, the AUC in a ROC model predicting significant CAD changed from 84% to 87% when adding P-OPG tertiles to the covariate predictive model that included P-NT-proBNP and CCS. Natural log-transformed P-OPG was also predictive for significant CAD (adjusted OR 3.08 [1.05-9.08], p = 0.041). Finally, the number of patients with >70% coronary artery stenosis were 2, 8 and 13 in the first, second and third P-OPG tertiles, respectively, and for patients with P-OPG in the highest (third) *vs. *first tertile, unadjusted and adjusted ORs of coronary artery stenosis were 7.56 (1.64-34.99) and 14.20 (1.35-148.92), respectively. Furthermore, among patients where CAG was performed (n = 86), 91% of the patients with P-OPG in the lower tertile (n = 22) had no >70% coronary artery stenosis (p = 0.030). However, in the study population as a whole the positive predictive value of high P-OPG for coronary artery stenosis was only 19%.

**Table 3:** The relations between plasma OPG and the prevalence of significant CAD as defined by abnormal MPI and/or stenosis on CAG.

Parameter	OR	95% CI	p-value
Study population as a whole (n = 200)	'Significant CAD'		
Unadjusted OPG (2^rd ^tertile vs. 1^st^)	2.74	1.23-6.10	0.013
Unadjusted OPG (3^rd ^tertile vs. 1^st^)	4.24	1.93-9.31	0.0001
Adjusted OPG (2^rd ^tertile vs. 1^st^)	3.51	1.39-8.86	0.008
Adjusted OPG (3^rd ^tertile vs. 1^st^)	3.54	1.36-9.21	0.010

Odds ratios (ORs) were assessed by logistic regression in univariate and multivariable analyses.

Covariate adjustments included variables associated with significant CAD in univariate analysis (p < 0.05), including P-total cholesterol, P-creatinine, peripheral systolic blood pressure, vibratory perception threshold and heart rate variability.

## Discussion

### Principal findings

The present study of 200 type 2 diabetic patients with microalbuminuria showed an independent predictive value of P-OPG for significant CAD, and although P-OPG correlated with P-NT-proBNP and CCS, its association with CAD remained significant after adjustment for these risk markers. Among patients with significant CAD (n = 70), 23 had >70% coronary artery stenosis at CAG and P-OPG was also independently associated with coronary artery stenosis. Among patients where CAG was performed, 91% of patients with low P-OPG did not have coronary artery stenosis at CAG.

### P-OPG as a biomarker for subclinical CAD

Several studies have demonstrated that P-OPG is a strong prognostic cardiovascular risk marker in diabetic and non-diabetic populations [2-5]. For example, we have found that in 283 type 2 diabetic subjects followed for 17 years, high P-OPG predicted all-cause and cardiovascular mortality, independently of known CVD risk markers including levels of albuminuria and P-NT-proBNP[2]. The latter study was not designed to examine mechanisms behind the increased P-OPG and cardiovascular death. In the current cross-sectional study, we demonstrated that P-OPG was a risk marker for the presence of subclinical CAD, which again may contribute to the association with CVD mortality. OPG expression is increased in the atherosclerotic arterial wall and P-OPG has been suggested to reflect the arterial OPG content and therefore be a surrogate marker of arterial damage[8]. Along this line, OPG is up-regulated in calcified coronary plaques[20] and P-OPG has been associated with angiographic disease severity, independent of conventional risk factors in other high risk populations[9,10]. One considerably smaller study (n = 40) has previously found that P-OPG was a risk marker for asymptomatic coronary artery stenosis in diabetic patients with at least one additional conventional risk factor[21]. In the present study, we confirm and extend this observation in a larger and well-defined high risk type 2 diabetic patient group with microalbuminuria. Specifically, the demonstrated association of P-OPG with CAD independent of NT-proBNP or CCS is new and this finding adds significantly to the current knowledge that P-OPG may be considered as an additional marker of atherosclerosis.

Several studies have demonstrated that micro- and macroalbuminura identifies a subgroup of diabetic patients with increased morbidity and mortality from CVD, but a recent study suggested that in the detection of subclinical disease additional markers are in need[22]. Both NT-proBNP levels and CCS are established markers of CVD mortality with high independent predictive capabilities in numerous populations[23,24]. Noteworthy, NT-proBNP levels and CCS in combination have synergistically predictive effect for CVD events in asymptomatic patients without history or signs of CAD followed for 3.9 years[25]. In contrast, other CVD markers fail to add prognostic value when compared with NT-proBNP and CCS, respectively[26,27]. It is therefore interesting in the present study, that P-OPG was independent of NT-proBNP and CCS and this adds to the body of evidence indicating that measurements of P-OPG might be useful as a biomarker for CAD. ROC analyses, however, revealed that the predictive accuracy of OPG alone was low and OPG only slightly improved the covariate model with NT-proBNP and CCS. Of note, the combined model with NT-proBNP and CCS without P-OPG already had an AUC of 0.84 and it is therefore difficult to increase the area further. Along this line, another study reported that P-OPG measurements alone are insufficient as markers of endothelial dysfunction[28]. Our study suggests that P-OPG could be used in combination with other markers. Previous studies that have used a multi biomarker approach have not included bone metabolism and P-OPG. Bone metabolism with P-OPG measurements may prove important markers of vascular calcification. Actually, CCS and P-OPG in combination could be particular interesting as markers of structural and functional vascular calcification, respectively. In the present study, P-OPG and CCS were related and 80% of patients with low P-OPG (<1^st ^tertile) also had CCS < 400. Furthermore, in patients with low P-OPG and/or CCS < 400, 83% of patients were also without CAD, however the positive predictive value was low (data not shown). Future studies may show that OPG in combination with CCS, NT-proBNP or other specific plasma markers may provide a strategy to identify patients with subclinical CAD.

If OPG is also a mediator of CVD is not known but OPG may influence plaque morphology and vulnerability through the increased expression of matrix metalloproteinases[29] Knowledge, however, concerning treatment effects on P-OPG and their possible link to clinical outcome is sparse. An earlier study has showed that low-dose simvastatin treatment for 18 weeks reduced P-OPG in type 2 diabetic patients with microalbuminuria and mild hypercholesterolemia[30]. Also, a recent study reported that 6 months of pioglitazone decreased P-OPG levels in type 2 diabetic patients[31]. However, in order to examine an effect on clinical outcomes, substantially longer study periods are of course needed, and a possible therapeutic benefit from lowering P-OPG remains to be shown in larger intervention studies.

### Clinical implications

Although it has important prognostic impact to demonstrate subclinical CAD, the therapeutic consequences of diagnosing asymptomatic CAD in diabetic patients remains to be shown. No randomized control trials have as yet examined if myocardial revascularization is beneficial in these asymptomatic patients, but in symptomatic diabetic patients receiving optimal medical therapy there was no difference between the groups receiving revascularization compared to controls[32].

Accordingly, the recommended primary and secondary CAD treatment remains implementation of intensified medical therapy aimed at reducing the conventional risk factors. The diagnosis of subclinical CAD is likely to encourage the efforts of both the patient and his/her physician to reduce conventional risk factors more effectively, which can diminish CVD morbidity as well as mortality in these patients[12].

### Limitations

As mentioned above, low (first tertile values) P-OPG excluded the presence of >70% coronary artery stenosis in 91% of the patients where CAG was performed according to our risk stratification algorithm. Although this was a high negative predictive value, there is obviously a significant selection bias in play between patients who had a CAG performed in the present study compared to an unselected group of patients with type 2 diabetes and microalbuminuria Furthermore, the positive predictive value of P-OPG for coronary artery stenosis was low. Therefore, the potential use of P-OPG as a rule-out marker needs confirmation in a larger, prospective study.

It is also notable, that we did not examine our 67 low risk patients for significant CAD, since low P-NT-proBNP and/or low CCS is associated with a very good prognosis in these patients[13]. In this study we therefore assumed in most of our analyses that these 67 patients were without significant CAD. We found, however, that P-OPG was predictive of CAD within the high risk group alone where all patients were investigated for CAD. Other study limitations included the relatively small sample size and that patients with elevated P-creatinine were excluded to allow for examinations with CT and CAG contrast media. Furthermore, all our patients with microalbuminuria were aggressively medically treated, i.e., the value of P-OPG in the diagnosis of asymptomatic CAD in less well-treated patients require further studies.

## Conclusion

Elevated P-OPG is an independent predictor of the presence of asymptomatic CAD in type 2 diabetic patients with microalbuminuria. The effect of P-OPG was independent of conventional CVD risk factors, including P-NT-proBNP and CCS. P-OPG may represent a simple test for excluding patients at high risk of subclinical CAD.

## Competing interests

Dr. Rossing reports having received lecture fees from Novartis and Boehringer Ingelheim, and research grant from Novartis, has served as a consultant for Merck, and having equity interest in NovoNordisk. Dr. Parving reports having served as a consultant for Novartis, Merck, Pfizer and Sanofi-Aventis, having equity interest in Merck and NovoNordisk and having received lecture fees from Novartis, Merck, Pfizer and Sanofi-Aventis. Dr. Parving has received grant support from Novartis, AstraZeneca and Sanofi-Aventis.

## Authors' contributions

H.R: researched data, contributed to discussion, wrote manuscript

PKJ, PR, MN, LM, R PRH, H-HP, NW, AK, CLP, KW: researched data, contributed to discussion, reviewed/edited manuscript.

All authors read and approved the final manuscript.

## References

[B1] Ninomiya T Perkovic V de Galan BE Zoungas S Pillai A Jardine M Patel A Cass A Neal B Poulter N Mogensen CE Cooper M Marre M Williams B Hamet P Mancia G Woodward M MacMahon S Chalmers J Albuminuria and kidney function independently predict cardiovascular and renal outcomes in diabetes J Am Soc Nephrol 2009 20 1813 1821 10.1681/ASN.2008121270 19443635 PMC2723977

[B2] Jorsal A Tarnow L Flyvbjerg A Parving HH Rossing P Rasmussen LM Plasma osteoprotegerin levels predict cardiovascular and all-cause mortality and deterioration of kidney function in type 1 diabetic patients with nephropathy Diabetologia 2008 51 2100 2107 10.1007/s00125-008-1123-8 18719882

[B3] Reinhard H Lajer M Gall MA Tarnow L Parving HH Rasmussen LM Rossing P Osteoprotegerin and mortality in type 2 diabetic patients Diabetes Care 2010 33 2561 2566 10.2337/dc10-0858 20929997 PMC2992191

[B4] Anand DV Lahiri A Lim E Hopkins D Corder R The relationship between plasma osteoprotegerin levels and coronary artery calcification in uncomplicated type 2 diabetic subjects J Am Coll Cardiol 2006 47 1850 1857 10.1016/j.jacc.2005.12.054 16682312

[B5] Nybo M Rasmussen LM The capability of plasma osteoprotegerin as a predictor of cardiovascular disease: a systematic literature review Eur J Endocrinol 2008 159 603 608 10.1530/EJE-08-0554 18697793

[B6] Simonet WS Lacey DL Dunstan CR Kelley M Chang MS Luthy R Nguyen HQ Wooden S Bennett L Boone T Shimamoto G DeRose M Elliott R Colombero A Tan HL Trail G Sullivan J Davy E Bucay N Renshaw-Gegg L Hughes TM Hill D Pattison W Campbell P Sander S Van G Tarpley J Derby P Lee R Boyle WJ Osteoprotegerin: a novel secreted protein involved in the regulation of bone density Cell 1997 89 309 319 10.1016/S0092-8674(00)80209-3 9108485

[B7] Emery JG McDonnell P Burke MB Deen KC Lyn S Silverman C Dul E Appelbaum ER Eichman C DiPrinzio R Dodds RA James IE Rosenberg M Lee JC Young PR Osteoprotegerin is a receptor for the cytotoxic ligand TRAIL J Biol Chem 1998 273 14363 14367 10.1074/jbc.273.23.14363 9603945

[B8] Olesen P Ledet T Rasmussen LM Arterial osteoprotegerin: increased amounts in diabetes and modifiable synthesis from vascular smooth muscle cells by insulin and TNF-alpha Diabetologia 2005 48 561 568 10.1007/s00125-004-1652-8 15700136

[B9] Jono S Ikari Y Shioi A Mori K Miki T Hara K Nishizawa Y Serum osteoprotegerin levels are associated with the presence and severity of coronary artery disease Circulation 2002 106 1192 1194 10.1161/01.CIR.0000031524.49139.29 12208791

[B10] Kiechl S Schett G Wenning G Redlich K Oberhollenzer M Mayr A Santer P Smolen J Poewe W Willeit J Osteoprotegerin is a risk factor for progressive atherosclerosis and cardiovascular disease Circulation 2004 109 2175 2180 10.1161/01.CIR.0000127957.43874.BB 15117849

[B11] Browner WS Lui LY Cummings SR Associations of serum osteoprotegerin levels with diabetes, stroke, bone density, fractures, and mortality in elderly women J Clin Endocrinol Metab 2001 86 631 637 10.1210/jc.86.2.631 11158021

[B12] Gaede P Lund-Andersen H Parving HH Pedersen O Effect of a multifactorial intervention on mortality in type 2 diabetes N Engl J Med 2008 358 580 591 10.1056/NEJMoa0706245 18256393

[B13] Reinhard H Hansen PR Persson F Tarnow L Wiinberg N Kjaer A Petersen CL Winther K Parving HH Rossing P Jacobsen PK Elevated NT-proBNP and coronary calcium score in relation to coronary artery disease in asymptomatic type 2 diabetic patients with elevated urinary albumin excretion rate Nephrol Dial Transplant 2011 10.1093/ndt/gfr009 21372253

[B14] Tarnow L Gall MA Hansen BV Hovind P Parving HH Plasma N-terminal pro-B-type natriuretic peptide and mortality in type 2 diabetes Diabetologia 2006 49 2256 2262 10.1007/s00125-006-0359-4 16937127

[B15] Lassen NA Tvedegaard E Jeppesen FI Nielsen PE Bell G Gundersen J Distal blood pressure measurement in occlusive arterial disease, strain gauge compared to xenon-133 Angiology 1972 23 211 217 10.1177/000331977202300405 5030556

[B16] Agatston AS Janowitz WR Hildner FJ Zusmer NR Viamonte M Jr Detrano R Quantification of coronary artery calcium using ultrafast computed tomography J Am Coll Cardiol 1990 15 827 832 10.1016/0735-1097(90)90282-T 2407762

[B17] Chiu YW Adler SG Budoff MJ Takasu J Ashai J Mehrotra R Coronary artery calcification and mortality in diabetic patients with proteinuria Kidney Int 2010 77 1107 1114 10.1038/ki.2010.70 20237457

[B18] Abbara S Arbab-Zadeh A Callister TQ Desai MY Mamuya W Thomson L Weigold WG SCCT guidelines for performance of coronary computed tomographic angiography: a report of the Society of Cardiovascular Computed Tomography Guidelines Committee J Cardiovasc Comput Tomogr 2009 3 190 204 10.1016/j.jcct.2009.03.004 19409872

[B19] Knudsen ST Foss CH Poulsen PL Andersen NH Mogensen CE Rasmussen LM Increased plasma concentrations of osteoprotegerin in type 2 diabetic patients with microvascular complications Eur J Endocrinol 2003 149 39 42 10.1530/eje.0.1490039 12824864

[B20] Dhore CR Cleutjens JP Lutgens E Cleutjens KB Geusens PP Kitslaar PJ Tordoir JH Spronk HM Vermeer C Daemen MJ Differential expression of bone matrix regulatory proteins in human atherosclerotic plaques Arterioscler Thromb Vasc Biol 2001 21 1998 2003 10.1161/hq1201.100229 11742876

[B21] Avignon A Sultan A Piot C Elaerts S Cristol JP Dupuy AM Osteoprotegerin is associated with silent coronary artery disease in high-risk but asymptomatic type 2 diabetic patients Diabetes Care 2005 28 2176 2180 10.2337/diacare.28.9.2176 16123486

[B22] Ito H Komatsu Y Mifune M Antoku S Ishida H Takeuchi Y Togane M The estimated GFR, but not the stage of diabetic nephropathy graded by the urinary albumin excretion, is associated with the carotid intima-media thickness in patients with type 2 diabetes mellitus: a cross-sectional study Cardiovasc Diabetol 2010 9 18 10.1186/1475-2840-9-18 20470427 PMC2877657

[B23] Di AE Chowdhury R Sarwar N Ray KK Gobin R Saleheen D Thompson A Gudnason V Sattar N Danesh J B-type natriuretic peptides and cardiovascular risk: systematic review and meta-analysis of 40 prospective studies Circulation 2009 120 2177 2187 10.1161/CIRCULATIONAHA.109.884866 19917883

[B24] Oudkerk M Stillman AE Halliburton SS Kalender WA Mohlenkamp S McCollough CH Vliegenthart R Shaw LJ Stanford W Taylor AJ van Ooijen PM Wexler L Raggi P Coronary artery calcium screening: current status and recommendations from the European Society of Cardiac Radiology and North American Society for Cardiovascular Imaging Int J Cardiovasc Imaging 2008 24 645 671 10.1007/s10554-008-9319-z 18504647 PMC2493606

[B25] Shaw LJ Polk DM Kahute TA Wong ND Moon J Miranda-Peats R Rozanski A Friedman JD Hayes S Thomson L Berman DS Prognostic accuracy of B-natriuretic peptide measurements and coronary artery calcium in asymptomatic subjects (from the Early Identification of Subclinical Atherosclerosis by Noninvasive Imaging Research [EISNER] study) Am J Cardiol 2009 104 1245 1250 10.1016/j.amjcard.2009.06.041 19840570

[B26] Blankenberg S McQueen MJ Smieja M Pogue J Balion C Lonn E Rupprecht HJ Bickel C Tiret L Cambien F Gerstein H Munzel T Yusuf S Comparative impact of multiple biomarkers and N-Terminal pro-brain natriuretic peptide in the context of conventional risk factors for the prediction of recurrent cardiovascular events in the Heart Outcomes Prevention Evaluation (HOPE) Study Circulation 2006 114 201 208 10.1161/CIRCULATIONAHA.105.590927 16831981

[B27] Folsom AR Kronmal RA Detrano RC O'Leary DH Bild DE Bluemke DA Budoff MJ Liu K Shea S Szklo M Tracy RP Watson KE Burke GL Coronary artery calcification compared with carotid intima-media thickness in the prediction of cardiovascular disease incidence: the Multi-Ethnic Study of Atherosclerosis (MESA) Arch Intern Med 2008 168 1333 1339 10.1001/archinte.168.12.1333 18574091 PMC2555989

[B28] Stepien E Wypasek E Stopyra K Konieczynska M Przybylo M Pasowicz M Increased levels of bone remodeling biomarkers (osteoprotegerin and osteopontin) in hypertensive individuals Clin Biochem 2011 44 826 831 10.1016/j.clinbiochem.2011.04.016 21539822

[B29] Zannettino AC Holding CA Diamond P Atkins GJ Kostakis P Farrugia A Gamble J To LB Findlay DM Haynes DR Osteoprotegerin (OPG) is localized to the Weibel-Palade bodies of human vascular endothelial cells and is physically associated with von Willebrand factor J Cell Physiol 2005 204 714 723 10.1002/jcp.20354 15799029

[B30] Nellemann B Gormsen LC Dollerup J Schmitz O Mogensen CE Rasmussen LM Nielsen S Simvastatin reduces plasma osteoprotegerin in type 2 diabetic patients with microalbuminuria Diabetes Care 2007 30 3122 3124 10.2337/dc07-0919 17804683

[B31] Park JS Cho MH Nam JS Yoo JS Ahn CW Cha BS Kim KR Lee HC Effect of pioglitazone on serum concentrations of osteoprotegerin in patients with type 2 diabetes mellitus Eur J Endocrinol 2011 164 69 74 10.1530/EJE-10-0875 20961967 PMC3000683

[B32] Frye RL August P Brooks MM Hardison RM Kelsey SF MacGregor JM Orchard TJ Chaitman BR Genuth SM Goldberg SH Hlatky MA Jones TL Molitch ME Nesto RW Sako EY Sobel BE A randomized trial of therapies for type 2 diabetes and coronary artery disease N Engl J Med 2009 360 2503 2515 19502645 10.1056/NEJMoa0805796 PMC2863990

